# Specific Electrogram Characteristics Identify the Extra-Pulmonary Vein Arrhythmogenic Sources of Persistent Atrial Fibrillation – Characterization of the Arrhythmogenic Electrogram Patterns During Atrial Fibrillation and Sinus Rhythm

**DOI:** 10.1038/s41598-020-65564-2

**Published:** 2020-06-04

**Authors:** Amir Jadidi, Mark Nothstein, Juan Chen, Heiko Lehrmann, Olaf Dössel, Jürgen Allgeier, Dietmar Trenk, Franz-Josef Neumann, Axel Loewe, Björn Müller-Edenborn, Thomas Arentz

**Affiliations:** 1 Department of Electrophysiology, University-Heart-Center Freiburg-Bad Krozingen, Bad Krozingen Campus, Germany; 2 Institute of Biomedical Engineering, Karlsruhe Institute of Technology, Karlsruhe, Germany; 3 Department of Clinical Pharmacology, University-Heart-Center Freiburg-Bad Krozingen, Bad Krozingen Campus, Germany; 4 Department of Cardiology, University-Heart-Center Freiburg-Bad Krozingen, Bad Krozingen Campus, Germany

**Keywords:** Interventional cardiology, Experimental models of disease

## Abstract

Identification of atrial sites that perpetuate atrial fibrillation (AF), and ablation thereof terminates AF, is challenging. We hypothesized that specific electrogram (EGM) characteristics identify AF-termination sites (AFTS). Twenty-one patients in whom low-voltage-guided ablation after pulmonary vein isolation terminated clinical persistent AF were included. Patients were included if short RF-delivery for <8sec at a given atrial site was associated with acute termination of clinical persistent AF. EGM-characteristics at 21 AFTS, 105 targeted sites without termination and 105 non-targeted control sites were analyzed. Alteration of EGM-characteristics by local fibrosis was evaluated in a three-dimensional high resolution (100 µm)-computational AF model. AFTS demonstrated lower EGM-voltage, higher EGM-cycle-length-coverage, shorter AF-cycle-length and higher pattern consistency than control sites (0.49 ± 0.39 mV vs. 0.83 ± 0.76 mV, p < 0.0001; 79 ± 16% vs. 59 ± 22%, p = 0.0022; 173 ± 49 ms vs. 198 ± 34 ms, p = 0.047; 80% vs. 30%, p < 0.01). Among targeted sites, AFTS had higher EGM-cycle-length coverage, shorter local AF-cycle-length and higher pattern consistency than targeted sites without AF-termination (79 ± 16% vs. 63 ± 23%, p = 0.02; 173 ± 49 ms vs. 210 ± 44 ms, p = 0.002; 80% vs. 40%, p = 0.01). Low voltage (0.52 ± 0.3 mV) fractionated EGMs (79 ± 24 ms) with delayed components in sinus rhythm (‘atrial late potentials’, respectively ‘ALP’) were observed at 71% of AFTS. EGMs recorded from fibrotic areas in computational models demonstrated comparable EGM-characteristics both in simulated AF and sinus rhythm. AFTS may therefore be identified by locally consistent, fractionated low-voltage EGMs with high cycle-length-coverage and rapid activity in AF, with low-voltage, fractionated EGMs with delayed components/ ‘atrial late potentials’ (ALP) persisting in sinus rhythm.

## Introduction

Pulmonary vein isolation (PVI) is currently the mainstay of therapy in patients with atrial fibrillation (AF). Arrhythmia freedom following PVI is high in paroxysmal AF, as pulmonary vein foci represent the main source of arrhythmia in these patients^1^. This is in contrast to persistent AF, where arrhythmogenic sources in the left-atrial body itself, and hence outside of the pulmonary veins, may contribute to the perpetuation of AF^2–4^. In this context, fibrotic remodeling of the atrial tissue is hypothesized to contribute to local development of micro-reentries or focal sources sustaining the arrhythmia^3,5^. As a consequence, arrhythmia freedom with PVI alone can be achieved in only about half of patients suffering from persistent AF^6,7^.

The importance of arrhythmogenic sources outside the pulmonary veins for perpetuation of persistent AF is supported by the fact that in 70% of these patients, local radiofrequency-ablation within the left atrium terminates ongoing AF following or even before PVI was performed^2–4^. The clinical challenge lies in the correct identification of these AF termination sites which is necessary for targeted ablation whilst avoiding unnecessary damage to healthy atrial myocardium.

For the current study, we hypothesized that AF termination sites share specific electrogram characteristics in AF and in sinus rhythm that would allow identification these sites for targeted ablation. A high-resolution computer-model including local fibrotic remodeling was used to investigate its impact on local electrogram characteristics during AF and sinus rhythm to mechanistically underpin the clinical observations.

## Methods

### Patient characteristics and ablation procedure

Twenty-one patients were included in the current study. They were consecutively recruited for a previously published study that investigated the impact of selective ablation of low-voltage-areas in AF in addition to circumferential PVI^8^. All patients analyzed in the current study had acute AF termination following <8 seconds of RF-energy-delivery at a given site. Inclusion criteria were symptomatic persistent AF (lasting >7 days and <12 months) and no previous right- or left-atrial ablations. Exclusion criteria were prior surgical or catheter ablation therapy or presence of atrial thrombus and major bleeding under oral anticoagulant therapy. The review board of the Medical Faculty of the University of Freiburg, Germany, approved the study and patients provided written informed consent. All methods were performed in accordance with the relevant guidelines and regulations.

Ablation was performed using an irrigated-tip contact-force sensor-enabled catheter (SmartTouch from Biosense Webster or TactiCath from Abbott) with 25 W for 20 seconds at the posterior LA wall and 30 W for 40 seconds at all other left atrial locations. Ablation terminated AF to atrial tachycardia (AT) in 15/21 and directly to sinus rhythm in 6/21 patients. If AF terminated into AT, all remaining ATs were ablated until sinus rhythm was achieved. Fractionated delayed electrograms in sinus rhythm within the 1 cm-wide border area of acute AF termination sites were also ablated.

### High-density atrial contact mapping

All 21 patients presented in clinical AF and underwent left-atrial mapping as described previously^3^ using a 20-pole catheter (AFocus II HD, Abbott) with 1 mm electrodes and 4-4-4 mm spacing, resulting in a simultaneous mapping field of 3.2 cm^2^ (in conjunction with the Ensite system, Abbott) or a 20-pole variable Lasso catheter (2-6-2 mm spacing) with the CARTO-V3 system (Biosense Webster). All bipolar recordings were included to create the bipolar voltage map of the atrium. To ensure highest accuracy of electrogram criteria, >1000 left-atrial sites were mapped. Only mapping sites within a distance of <5 mm from the atrial geometry were considered for the voltage map, in order to avoid inclusion of electrograms recorded at electrodes without contact with atrial tissue.

### Electrogram criteria defining target sites for ablation

**Figure 1:**
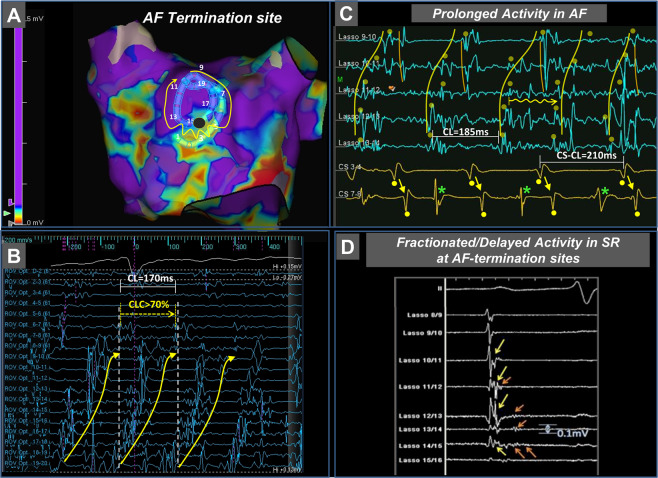
Electrogram Characteristics at Atrial Fibrillation Termination Sites. Atrial fibrillation terminated after local radiofrequency delivery of 5 seconds at a low voltage-site with bipolar voltage <0.5 mV (maroon marker, **A**). Electrograms recorded from this site using a 20-polar circumferential catheter demonstrate spatio-temporal dispersion of fractionated electrograms spreading over 14 bipoles and covering more than 70% of the AF cycle length (white markers give total AF cycle length; yellow marker gives the cycle length covered by spatio-temporal dispersion. Electrodes 12–16 display prolonged low voltage activity (**B**). Prolonged fractionated activity covering ≥70% of the AF cycle length at given a site (on 1–2 adjacent bipoles). Also mark that the local AF cycle length (blue EGMs) is faster than the concomitant AF cycle length in the coronary sinus (yellow EGMs). The local AF cycle length was determined using morphological electrogram criteria, combined with regional activation pattern recognition of neighboring electrodes (e.g. yellow dots on Lasso 10–11 detect qs-shaped sharp electrogram component that is observable during every AF beat; yellow curve annotates the shape of the earliest electrograms on the Lasso electrodes; mean AF cycle length was computed from 10 consecutive AF intervals, **C**, and Supplemental Fig. 1). Also mark that EGMs on CS7-8 show two distinctive wave fronts: One type with smooth far-field Qr-morphology (yellow dots below them and yellow arrows), which reflect the same activity/wave front as on CS 3–4, and a second distinct near-field wave front with sharper electrograms and Rs-morphology (green asterisks). These wave fronts likely represent far field endocardial depolarizations and local intra-CS electrograms, respectively (**C**). Electrograms recorded from the imminent vicinity of the ablation lesion following termination of AF to sinus rhythm show fractionated (yellow arrows) and delayed (orange arrows) low-voltage electrogram components (corresponding to ‘atrial late potentials’ (ALP)) as the underlying slow conduction substrate at the site of AF driver (**D**). AF, atrial fibrillation; CL, cycle length; CLC, cycle length coverage.
II. Electrical activity covering ≥70% of the AF cycle length at a site (prolonged fractionated activity on 1–2 adjacent bipoles, Fig. 1C)
III. Local AF cycle length averaged over ten consecutive AF beats on a given bipolar electrode and AF cycle length concomitantly measured in the coronary sinus. Rapid local activity was diagnosed when local AF cycle length was ≥15% shorter than the concomitant AF cycle length in the coronary sinus (Fig. 2A,B; figure 1C and see supporting informations in supplemental figure 1 for AFCL measurement).

Electrogram criteria defining target sites were established during AF as described previously^3^. Low voltage areas were delineated based on a bipolar voltage <0.5 mV during AF (peak-to-peak voltage of 1 beat in CARTO and 1-3  consecutive AF beats in Ensite, with QRS-exclusion). High mapping density (2–3 acquisitions per site) integrated voltage variations that occur in AF. We used the following additional criteria to define a potential target site for ablation:

I. Electrogram patterns recorded within low-voltage areas and their 1cm-wide border zone showing a sequential activation on the circumferential mapping catheter that exceeded 8 electrodes (>210°) and covered ≥70% of the local AF cycle length were considered as sites with spatio-temporal dispersion (Fig. 1A,B). These sites were targeted if spatio-temporal dispersion was observed in more than 3 of 10 consecutive AF beats.

**Figure 2:**
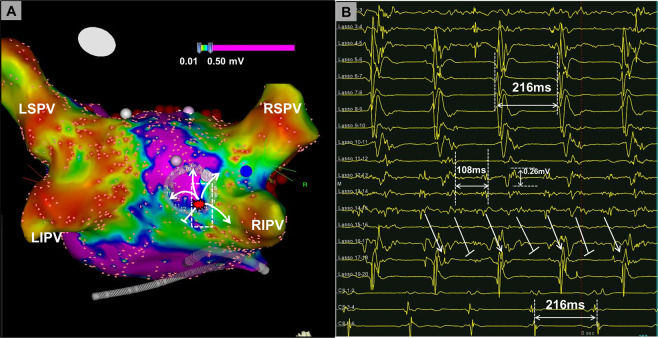
Rapid Focal Fibrillatory Sources. Shown is the voltage map (recorded in AF) of a patient who had a rapid focal source located in the border zone of a low-voltage area <0.5 mV (**A**). A 20-pole circumferential catheter demonstrates rapid activity with, low-voltage electrograms displaying a local cycle length of 108 ms, with a 2:1 conduction to the adjacent atrium (resulting in left atrial and coronary sinus-cycle length of 216 ms, **B**). Focal pressure with catheter tip interrupted the focal rapid activity, but the activity recovered after reduction of catheter tip contact. Focal ablation at this site terminated AF within 3 seconds of radiofrequency delivery. AF, atrial fibrillation; LSPV, left superior pulmonary vein; LIPV, left inferior pulmonary vein; RSPV, right superior pulmonary vein; RIPV, right inferior pulmonary vein.

### Analysis of AF termination sites

All AF termination sites and their 1cm-wide border zones were analyzed in AF (pre-ablation) and again following sinus rhythm restoration. The electrogram patterns recorded from these areas were assessed for bipolar voltage, longest electrogram duration, cycle length, cycle length coverage (percentage of the local AF cycle length that is covered by the mapping field of the circumferential catheter), spatio-temporal dispersion and consistency of prolonged activity (calculated as percentage of 10 consecutive AF beats at a given site with prolonged activity).

These electrogram characteristics were then compared to electrograms recorded from a total of 105 targeted sites (five sites in the left atrium in each of the 21 study patients) at which ablation did not terminate AF, and to electrograms at 105 evenly distributed non-targeted left atrial sites.

After sinus rhythm restoration, the acute AF termination site and its 1 cm-wide non-ablated border zone were remapped using the 20-pole circumferential catheter or the ablation catheter and analyzed for bipolar voltage and fractionated or locally delayed activation displaying atrial late potentials (‘ALP’) (Fig. 1D).

### Computer simulation of atrial fibrosis in atrial fibrillation and sinus rhythm in a high-resolution model

A stable rotational source was generated by cross field stimulus protocol in an isotropic virtual tissue patch measuring 30 mm × 30 mm × 2 mm with a spatial resolution of 100 µm (0.1 mm × 0.1 mm × 0.1 mm). Membrane kinetics were represented by the Courtemanche *et al*. model^9^ adapted to mimic persistent AF conditions^10^. The conductivity of the non-fibrotic region was adapted to yield a plane wave conduction velocity of 0.8 m/s and a peak-to-peak voltage of 1 mV. Regional fibrosis of was incorporated into the model within an area of 10 mm × 10 mm × 2 mm at the center of the patch and was modeled by setting the conductivity for fibrotic elements to zero. Since the exact distribution of fibrosis is unknown, we considered a Gaussian distribution of trans-mural 200 µm × 200 µm non-conductive elements within the fibrotic area affecting 40% of elements. The size of non-conductive elements was extrapolated from histological studies on collagen septa in aging human atria^11^. Excitation propagation was simulated using the cardiac electrophysiology solver acCELLerate^12^. Extracellular potentials were sampled with 1 kHz.

### Statistical analysis

Statistical analysis was performed using IBM SPSS Statistics 20 (IBM Corporation, Armonk, NY) and Graphpad Prism 7.0 (Graphad Software, San Diego, CA; www.graphpad.com/scientific-software/prism/). Data were checked for normal distribution by visual assessment of histograms and q–q plots. Normally distributed data are presented as mean ± SD and intergroup differences calculated with t-test or ANOVA with Bonferroni-post hoc correction, as appropriate. Non-normally distributed variables are given as median and interquartile range, with differences calculated using Mann-Whitney-U test or Dunn’s multiple comparison test. For all analyses, a p-value of ≤0.05 was considered significant.

## Results

### Localization of atrial fibrillation termination sites and their spatial relationship to low voltage areas

Table 1 gives the clinical characteristics of study patients. The majority of patients were male (14/21), had preserved left ventricular function (18/21) and received antiarrhythmic medications (13/21). After completion of circumferential isolation of all pulmonary veins (ablation-time for PVI: 27 ± 6 min), all 21 patients underwent low voltage-guided ablation with the aim to achieve AF termination. Acute AF termination was achieved in all patients after 8 ± 7 min of radiofrequency-application to LA low voltage areas (<0.5 mV in AF).

**Table 1:** Clinical characteristics of Study Patients.

	Total (n = 21)
Male sex (%)	14 (67)
Age (y)	63 (7)
LVEF (%)	58 (4)
Left ventricular dysfunction (LVEF<50%) (n)	3 (14)
LVEDD (mm)	51 (5)
IVS (mm)	11 (3)
LA diameter (mm)	44 (4)
Structural Cardiomyopathy (n)	6 (28)
Coronary Artery Disease (n)	2 (9)
Hypertension (n)	13 (62)
Mitral Regurgitation	mild: 1 (5)
	moderate: 2 (9)
History of Cerebral Ischemic Event (n)	3 (14)
Antiarrhythmic therapy (n)	Amiodarone: 8 (38)
	Dronedarone: 1 (5)
	Sotalol: 1 (5)
	Flecainide: 3 (14)

Values are given as n (%) or mean with standard deviation.

LA, left atrium; IVS, interventricular septum; LVEDD, left ventricular end-diastolic diameter; LVEF, left ventricular ejection franction.

**Figure 3:**
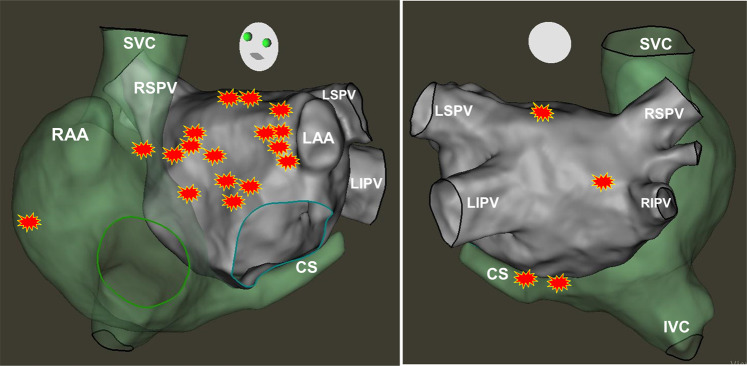
Anatomical distribution of acute AF termination sites in all 21 patients. All AF termination sites were located within low voltage <0.5 mV (in AF) areas. Most AF termination sites were located at the antero-septal LA and anterior base of the left atrial appendage (10/21 and 5/21, respectively). In two cases AF termination occurred proximal to the pulmonary vein antrum, two further occurred within the proximal and mid coronary sinus and the two remaining AF terminations occurred at the lateral wall of the right atrium (crista terminalis area) and the superior portion of the right atrial antero-septum (between SVC and RAA).

The majority of AF termination sites were located in the anteroseptal- or roof-region of the LA. Only two AF termination sites were located in the coronary sinus (Fig. 3), two at the posterior LA and one at the lateral right atrium (involving the crista terminalis).

Ninety percent of ablation sites (19/21) that lead to acute termination of AF were located within areas displaying a bipolar voltage of <0.5 mV in AF. In the remaining 10% (2/21), AF termination sites were found within a 1cm-wide border zone between low-voltage areas <0.5 mV and areas with sustained voltage >0.5 mV.

### Electrogram characteristics of atrial fibrillation termination sites

#### Electrogram voltage, duration, cycle length coverage and consistency of prolonged activity in atrial fibrillation

**Figure 4:**
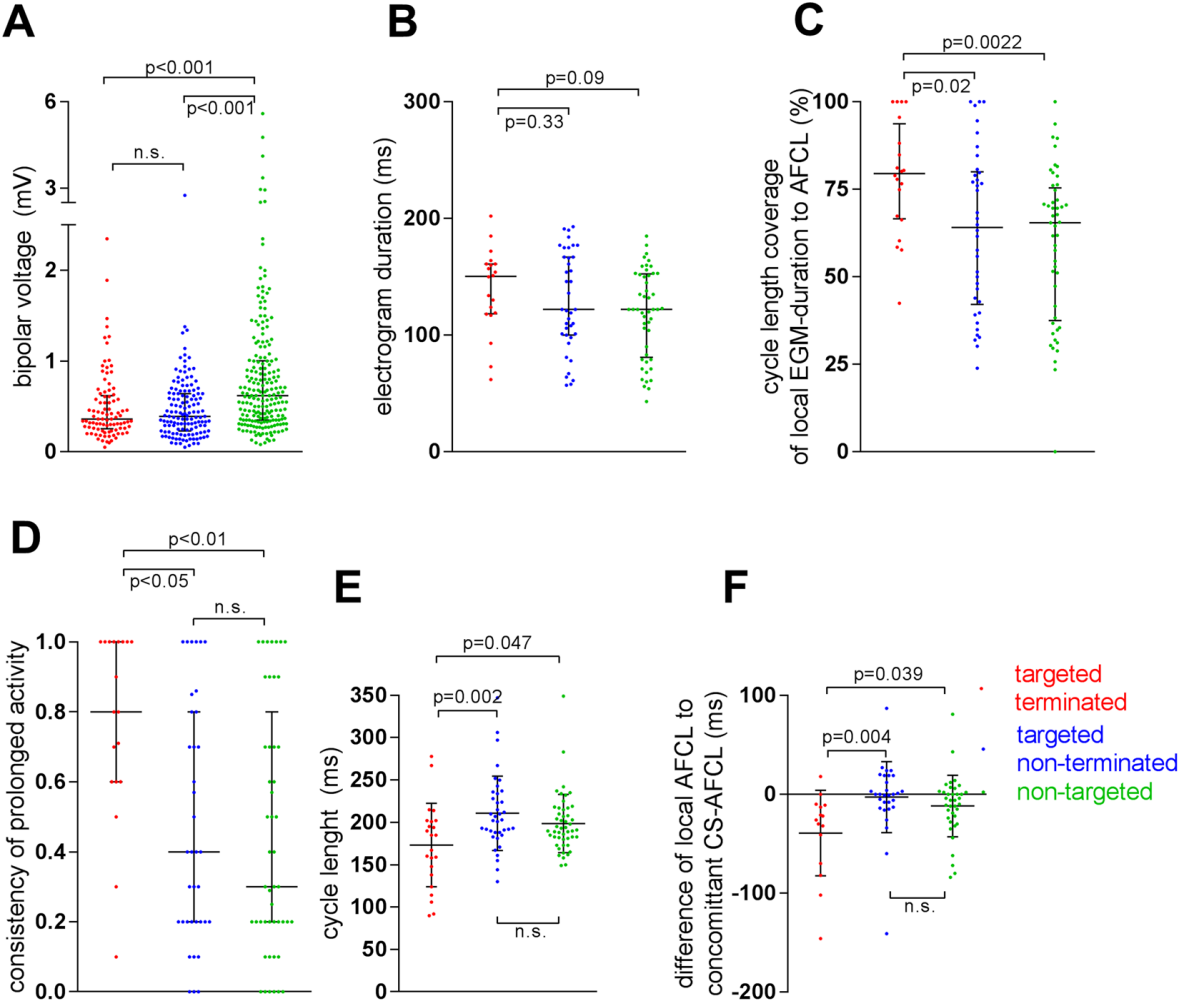
Bipolar Voltage, Electrogram Duration, Cycle Length Coverage, Consistency of Prolonged Activity and AF cycle length To Define Atrial Fibrillation Termination Sites. Sites targeted for ablation (ablation sites with successful termination of AF in red; sites at which ablation did not terminate AF in blue) show lower bipolar voltages than randomly selected and non-targeted control sites (in green, **A**). Successful termination sites (in red) do not differ significantly in electrogram duration from both ablated but non-successful sites (in blue) and non-targeted control sites (in green, **B**). Cycle length coverage given as percentage of local AF cycle length covered by the local EGM-duration, is high at successful termination sites (in red) and significantly lower at unsuccessful ablation sites (in blue) and non-targeted control sites (in green, **C**). The consistency of prolonged electrograms (given as percentage of ten consecutive AF-beats at a given site with prolonged electrograms) was higher in successful termination sites (in red, **D**). Absolute AF cycle length was shorter at successful termination sites (in red) as compared to targeted sites at which ablation did not terminate AF (in blue) and randomly selected non-targeted control sites (in green, **E**). The local cycle length, was significiantly shorter than the concomitant cycle length in the coronary sinus at succesful termination sites (in red), but not at ablation sites without termination (in blue) and non-targeted control sites (in green, **F**). AFCL, atrial fibrillation cycle length; CS-AFCL, atrial fibrillation cycle length in the coronary sinus.

Compared to 105 randomly selected non-targeted sites that were evenly spread within the left-atrial body of the 21 patients, AF termination sites were characterized by a lower local bipolar voltage (0.83 ± 0.76 mV vs. 0.49 ± 0.39 mV, p < 0.0001, Fig. 4A). Despite electrogram fractionation, a trend for longer absolute electrogram-duration at successful termination sites did not reach significance (139 ± 35 ms vs. 118 ± 37 ms, p = 0.09 m Fig. 4B). However, electrograms at termination sites covered a significantly larger part of the local AF cycle length as compared to control sites (cycle lengths coverage was 79 ± 16% at termination sites vs. 59 ± 22% at control sites, p = 0.0022, Fig. 4C) and demonstrated higher consistency of prolonged activity over 10 consecutive AF beats (0.8 [0.6/1.0] vs. 0.3 [0.2/0.8], p < 0.01; Fig. 4D, respectively).

Comparison of electrograms at successful AF termination sites to targeted sites at which ablation did not terminate AF, revealed comparable electrogram voltage (0.49 ± 0.39 mV vs. 0.48 ± 0.35 mV, p = 1.0; Fig. 4A) and electrogram duration (139 ± 35 ms vs. 129 ± 42 ms, p = 0.33; Fig. 4B). However, both cycle length coverage (79 ± 16% vs. 63 ± 23%, p = 0.02, Fig. 4C) and the consistency of prolonged activity were significantly higher at successful AF termination sites vs. ablation sites without impact on the fibrillatory activity (0.8 [0.6/1.0] vs. 0.4 [0.2/0.8], p = 0.01; Fig. 4D).

#### Electrogram cycle length at AF termination sites compared to other left atrial sites

Successful AF termination sites demonstrated shorter AF cycle lengths as compared to targeted sites without termination (173 ± 49 ms vs. 210 ± 44 ms, p = 0.002) and to non-targeted control sites (173 ± 49 ms vs. 198 ± 34 ms, p = 0.047; Fig. 4E).

Importantly, the cycle length at successful AF termination sites was also significantly shorter than the concomitant cycle length in the coronary sinus (by −39 ± 43 ms at AF termination sites vs. −3 ± 35 ms at unsuccessful targeted sites and −12 ± 31 ms at non-targeted control sites, p < 0.05; Fig. 4F). A representative sample of the mean cycle length measurement is demonstrated at different atrial locations in Supplemental Fig. 1.

#### Rapid focal activity at AF termination sites and relationship to low voltage

Rapid focal activity with a local AF cycle length more than 15% shorter as compared to the concomitant AF cycle length in the coronary sinus was found at 7 of 21 AF termination sites. Figure 2A,B demonstrates one of these rapid focal sources originating from the posterior left atrium near the right inferior pulmonary vein antrum with a 2:1 conduction to the adjacent atrium, coronary sinus and the pulmonary veins. The rapid fibrillatory source displays low voltage (≤0.26 mV; Fig. 2B, Lasso electrodes 12–15) electrograms with prolonged activity. Notably, on the CARTO voltage map which interpolates the voltage values of mapping points, the rapid source projects to the border zone <0.5 mV.

#### Electrogram characteristics of af termination sites in sinus rhythm

**Figure 5:**
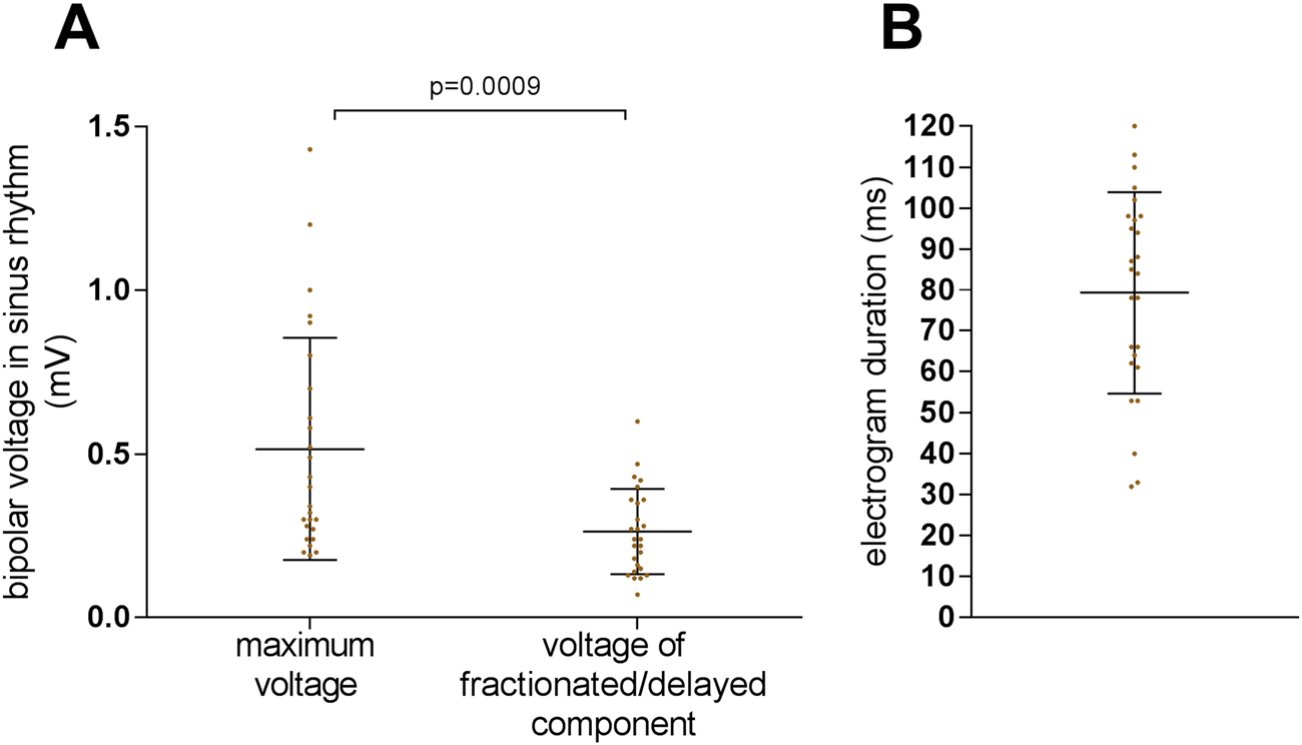
Electrogram Characteristics in Sinus Rhythm at Successful Termination Sites. Electrograms recorded in the imminent vicinity (within 1 cm) of successful AF termination sites were reevaluated following sinus rhythm restoration for maximum bipolar voltage (usually the first component in case of fractionated delayed electrograms and voltage of the fractionated delayed electrogram itself) (**A**) and duration of the total electrogram (**B**).

All AF termination sites were reassessed in sinus rhythm following termination. In 15/21 (71%) patients, the non-ablated, 1cm-wide border zone around the ablation site demonstrated low voltage-electrograms <0.5 mV with delayed fractionated components that correspond to ‘local late potentials’ (yellow and orange arrows in Fig. 1D**)**. The maximum bipolar voltage of all AF termination sites was 0.52 ± 0.3 mV, followed by a significantly lower voltage of its delayed fractionated components/ ‘atrial late potentials (ALP)’ (0.26 ± 0.13 mV, p = 0.0009; Fig. 5A [yellow and orange arrows in Fig. 1D]). Electrogram duration at these sites measured 79 ± 24 ms (Fig. 5B).

#### Mechanistic insights from computer simulation: heterogeneously distributed non-conductive elements simulating regional myocardial fibrosis are associated with stabilization of rotational sources in a 3d-model of atrial fibrillation

**Figure 6:**
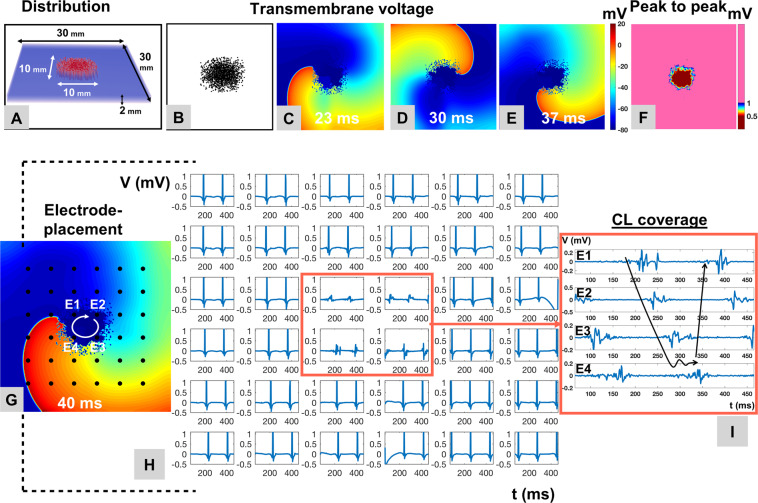
Stabilization of Localized Reentrant Activity with Electrogram Fragmentation in Atrial Fibrillation in a Three-Dimensional AF-Model with regional Atrial Fibrosis. A three-dimensional model incorporating a fibrotic patch (10 × 10 × 2 mm, spatial resolution of 100 µm) constituted of 40% non-conductive elements in a Gaussian distribution served to simulate the impact of atrial fibrosis on maintenance of rotational activity in an AF model (**A**,**B**). A stable rotational source was generated by cross field stimulation. Trans-membrane voltage maps show anchoring and stabilization of a reentrant rotational source around the simulated fibrotic area (**C–E**). The fibrotic area displays low peak-to-peak bipolar voltage in AF (**F**). A 6 × 6 electrode arrangement with inter-electrode distances of 2 mm was overlaid (**G**). Local electrograms recorded over the rotational source and within the fibrotic display low voltage and prolonged fractionated activity with a coverage of >70% of the AF cycle length at the center of the rotational source (**H**,**I**).

The impact of heterogeneously distributed non-conducting elements as a correlate of the low-voltage areas observed in study patients was evaluated in a computer model. Atrial fibrillation was first induced in a model that contained a simulated fibrotic area of 10 mm × 10 mm × 2 mm (Fig. 6A,B). In this model, the wave front invaded the affected area and anchored to it, leading to development of a stable rotational source displaying rotational electrical activity around the fibrotic area (Fig. 6C–E). Simulated electrograms recorded from within this area showed low bipolar voltage (Fig. 6F) with prolonged duration that covered ≥70% of the simulated AF cycle length with spatio-temporal dispersion pattern on virtual electrodes E1-4 (Fig. 6G–I).

**Figure 7:**
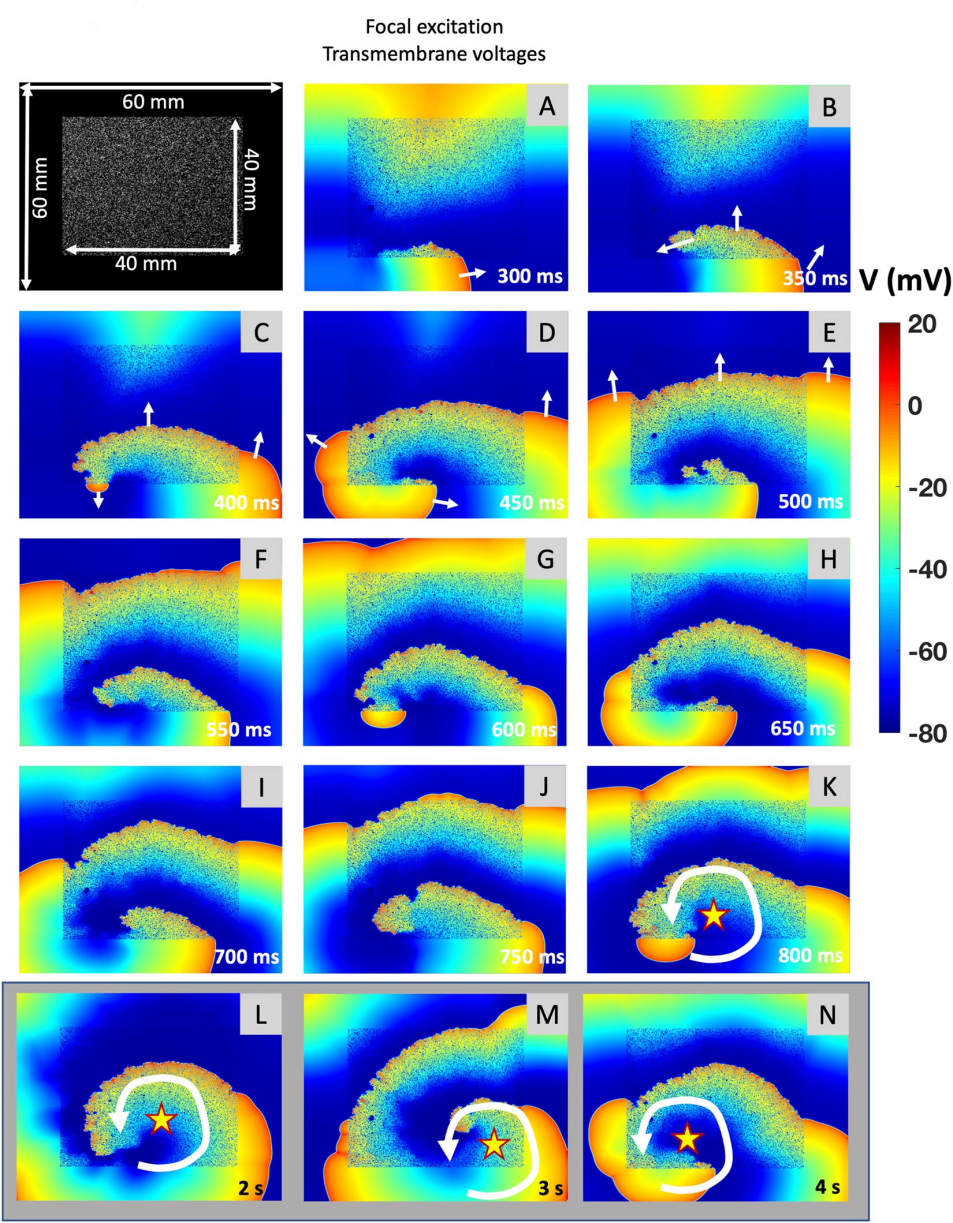
Multiple Wavelet Formation with Large Simulated Fibrosis in Atrial Fibrillation. Transmembrane voltage of a fibrotic tissue simulation of a meandering re-entry circuit. The top left picture shows the patch dimensions; white speckles indicate fibrotic tissue modeled as non-conductive elements. Elements (**A–K**) are equidistantly distributed in time (Δt = 50 ms). After cross field-stimulation, the depolarization wave propagates outside of fibrotic tissue (**A**). The depolarization wave then enters into the fibrotic tissue and continues propagation outside the fibrotic region (**B**). The depolarization wave exits the fibrotic region in (**C**). This pattern occurs repeatedly (**D–K**). The reentrant activity is marked with a circular arrow; the yellow star marks the center of rotation (**K**). Larger time intervals (Δt = 1 s) showing the long-term meandering propagation of the rotor and its center throughout the fibrotic tissue is shown in (**L,M**).

Increasing the size of the fibrotic area to 40 mm × 40 mm × 2 mm (Fig. 7A) again lead to anchoring of the wave front to the affected area. In addition, the wave front split into multiple wavelets within the fibrotic area which exited the area at different sites propagating in different directions (Fig. 7B–J). Moreover, a localized reentry with variable focal exit sites from the border zone of the fibrotic area was observed (Fig. 7K–N).

The effect of focal ablation within fibrotic areas demonstrating continuous activation in AF is demonstrated in Supplemental Fig. 2A–N. A single ablation point with a diameter of 2 mm shortens the self-sustaining fibrillatory activity (Supplemental Fig. 2I–K); a second ablation point terminates AF permanently (Supplemental Fig. 2L,M).

#### Heterogeneously distributed non-conductive elements simulating regional myocardial fibrosis affect electrogram characteristics in simulated sinus rhythm

**Figure 8:**
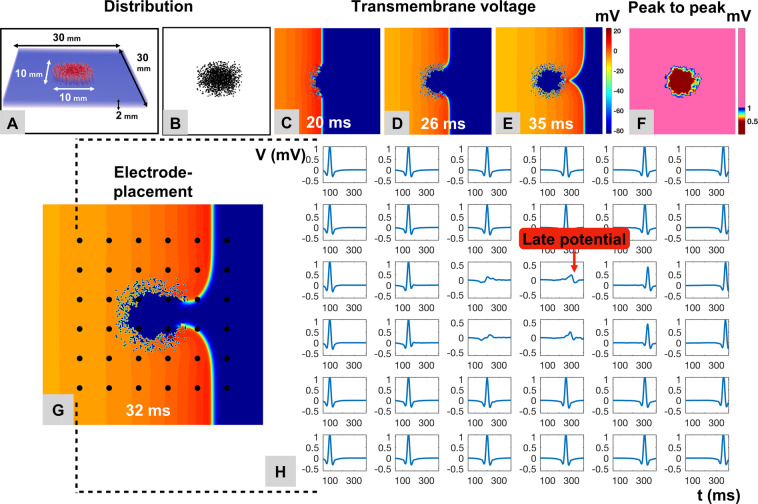
Development of Fractionated Delayed Electrogram Components in Simulated Sinus Rhythm. A single uniformly propagating wave front to emulate sinus rhythm was used in a three-dimensional model simulating atrial fibrosis as described in for Fig. 5 (**A**,**B**). Upon striking the simulated fibrotic area, the wave front warps concavely due to the change of the local source-sink relation and reduced conduction velocity (**C–G**). Local electrograms recorded within the fibrotic area demonstrate low bipolar voltage (**F**) and late potentials (**H**).

Using the smaller fibrotic area (10 mm × 10 mm), sinus rhythm was simulated with a single uniformly propagating wave front (Fig. 8A–E). The wave front was concavely warped when hitting the fibrotic area due to the change of the local source-sink relation and reduced conduction velocity (Fig. 8C–G). Local electrograms recorded within the fibrotic area demonstrated low bipolar voltage (Fig. 8F) and late potentials (Fig. 8H).

## Discussion

We report three main findings that help to identify non-pulmonary vein arrhythmic sources of human atrial fibrillation and support the importance of local fibrotic remodeling in the perpetuation of persistent atrial fibrillation:

First, AF termination sites are very closely spatially associated to low-voltage areas, with 90% of them located within areas demonstrating a bipolar voltage <0.5 mV in AF, and the remaining 10% located in the immediate border zone around such areas.

Second, the local electrograms at AF termination sites demonstrate a combination of low local bipolar voltage, high cycle length coverage, short local AF cycle length and high consistency of these electrogram characteristics over multiple AF beats.

Third, the surrounding zone of AF termination sites also demonstrates pathologic electrogram characteristics during sinus rhythm, with the majority of electrograms displaying low-voltage, fractionated and delayed electrogram components, with ‘atrial late potentials (ALP)’, supporting ablation of such sites in sinus rhythm for elimination of the underlying arrhythmogenic slow conduction substrate.

## Specific electrogram characteristics at atrial sites that perpetuate atrial fibrillation ***In Vivo*** and ***In Silico***

### Electrogram voltage

Fibrotic remodeling of previously healthy left atrial tissue contributes to the perpetuation of persistent AF. In this context, Nademanee and Haissaguerre *et al*. were the first to demonstrate that an arrhythmogenic source located within the left atrial body is sufficient to sustain AF, as its ablation terminates AF^13,14^.

Our current study demonstrates that sites associated with AF termination are not randomly distributed in the left atrium, but localized either within or in the immediate border zone of fibrotic areas with pathologically decreased local bipolar voltage <0.5 mV in AF. Targeted electrograms with successful termination therefore had bipolar voltages of 0.49 ± 0.39 mV in AF.

An important reduction of the electrical wave front amplitude by more than 80% upon entering the simulated fibrotic area was also found in the computer model used in our study, again resulting in simulated bipolar electrogram voltages <0.5 mV.

These findings are supported by clinical trials demonstrating that ablation of low-voltage areas, in addition to PVI, decreases arrhythmia recurrence in persistent AF^3,15,16^. They are also in line with studies reporting that the majority of left atrial sites driving AF (identified by panoramic non-invasive mapping and computational modeling) are located in or at the borders of fibrotic areas identified by MRI^17,18^.

While all successful termination sites demonstrated low-voltage electrograms, this characteristic was not exclusive and also found in targeted sites that did not lead to AF termination. Isolated low bipolar amplitude may therefore be considered a sensitive, yet not highly specific criterion for sites that perpetuate AF, which should prompt further analysis of the potential target electrogram. Adequate signal amplification can be considered a prerequisite for proper identification of additional electrogram characteristics such as fractionated or delayed components.

### Electrogram duration, cycle length coverage and consistency

Compared to electrograms at healthy atrial areas with preserved bipolar voltages, those recorded from targeted low-voltage areas showed fractionation, but the absolute electrogram duration did not differ significantly between successful termination sites and other sites (Fig. 4B). Instead, we found that cycle length coverage, that is the percentage of local AF cycle length covered by fractionated/prolonged electrograms (within a 2–3 cm^2^ measuring mapping area), was exceedingly high at successful termination sites (Fig. 4C) and showed high consistency (80%; 8 of 10 consecutive AF beats; Fig. 4D). This suggests underlying localized reentry- or repetitive focal activity with anisotropic and/or inhomogeneous conduction to surrounding myocardium at the given activation rates.

In our simulation model, heterogeneous conduction in simulated fibrotic areas was able to support localized reentries (Fig. 6). The simulated electrogram patterns recorded from within these localized reentries demonstrated locally consistent and repetitive prolonged electrograms, similar to our clinical observations. Observation of these electrograms characteristics at potential target site may therefore be due to narrow slow conduction isthmuses in the reentrant path, which also would explain the success of local ablation.

### Local cycle length and rapid focal triggers/drivers

Electrograms at AF termination sites displayed significantly shorter mean local cycle lengths (173 ± 49 ms) than unsuccessful ablation sites (210 ± 44 ms) or non-targeted control sites (198 ± 34 ms**;** Fig. 4E). Successful termination sites also displayed shorter local cycle lengths than the concomitant AF cycle length within the coronary sinus (by −39 ± 43 ms**;** Fig. 4F), which was not seen at unsuccessful ablation sites or non-targeted control sites. One third of patients met our predefined criteria for rapid focal activity (that is a local AF cycle length shorter than 15% than the concomitant cycle length in the coronary sinus) at their successful termination site. Local AF cycle length can therefore be considered an important criterion to identify arrhythmogenic sources of atrial fibrillation, which should be considered for the development of novel mapping algorithms.

### Electrogram properties in sinus rhythm

Characteristic electrogram properties with low-voltage, fractionation and delayed components with ‘atrial late potentials (ALP)’ were found during sinus rhythm in the immediate (and non-ablated) surroundings of successful AF termination sites in most (71%) cases (Fig. 5A,B). Using a single uniformly propagating activation wave front to simulate sinus rhythm in our computer model, we again found a high resemblance of electrograms recorded from within the simulated fibrotic area to the clinical electrograms. This suggests that the electrogram characteristics observed in fibrotic areas during AF are not functional and only due to the rapid fibrillatory activity, but rather specific to the underlying atrial substrate with locally delayed conduction within the slow conducting fibrotic areas.

### Simulation of fibrosis in atrial fibrillation and sinus rhythm

Previous computer models and lower-resolution MRI-based models demonstrated that heterogeneous conduction as found *in vivo* in areas of sub-total atrial fibrosis can support localized re-entries and disintegrate a regularly propagating electrical wave front as in sinus rhythm into irregular patterns as observed in AF^19–21^.

Our computer simulations differ from these previous models as they are based on an exceedingly high local resolution (100 µm^3^) that reaches the microscopic level in histological terms. The data derived from this model confirm previous findings and allow a detailed analysis of electrogram patterns in simulated fibrosis that explain the electrogram characteristics at clinical AF termination sites. The simulated fibrotic sites exhibit similar behavior as observed clinically at AF termination sites, with decreased local voltage and consistently prolonged electrical activity at the source of rotational activity.

### Technical considerations in the clinical setting

The EGM characteristics described in the current study contain in the majority of cases low (<0.5 mV) or very low (0.05–0.25 mV) voltage components, which are crucial for the identification of the arrhythmogenic AF sources. This emphasizes the technical standards that are required to analyze such EGMs with reasonable sensitivity and reproducibility. Any noise filtering, apart from standard 0.05–250 Hz low-/high pass filter, should be avoided and instead high quality, low-noise wiring be used. In order to avoid motion artifacts, we keep the mapping catheter at each atrial position for 10 to 15 seconds, allowing to assess consistency and search for the above-mentioned electrogram patterns.

### Limitations

A general limitation that is inherent to clinical mapping studies is that endocardial contact mapping is sequential, mapping one atrial site that is the size of the circumferential catheter (approximately 3.2 cm^2^) at a time^3,4,22^. Basket-catheters can increase the simultaneously mapped atrial area, but suffer from uneven distribution of only 64 electrodes with few electrodes having sufficient tissue contact and an overall low spatial mapping resolution.

Current computer models of AF on the other hand, are based on human action potential kinetics that were observed in cardiomyocytes from patients with AF^9^. While our model, in contrast to previously employed models, uses a spatial resolution comparable to the physiological scales in human atrial fibrosis, it does not incorporate all the molecular and cellular changes that develop in an aging human heart exposed to AF.

Finally, we do not know the exact voltage threshold to be considered pathological in AF or sinus rhythm. Some groups emphasize bipolar cut off-values as low as <0.24 mV in AF, roughly corresponding to <0.5 mV in sinus rhythm^23^. We use a cut off of <0.5 mV in AF and <1.0 mV in sinus rhythm^3^, as fragmented and prolonged electrograms in AF and delayed activity with ‘atrial late potentials (ALP)’ in sinus rhythm are in our experience and that of others frequently found within these voltages^24^, when mapping includes all bipole recordings (both the small and large spaced bipoles) of the 20-polar Lasso catheter.

## Conclusions

Successful termination sites of AF display distinct electrogram patterns with short local cycle lengths harboring fractionated and low-voltage potentials that are locally highly consistent and cover a majority of the local AF cycle length. Most of these areas also display pathologic delayed ‘atrial late potentials (ALP)’ and fractionated electrograms in sinus rhythm.

## Supplementary information

Supporting  movie 1A.

Supporting movie 1B.

Supporting movie 1C.

Supporting information.
