# Supplementary material for: Specific Electrogram Characteristics Identify the Extra-Pulmonary Vein Arrhythmogenic Sources of Persistent Atrial Fibrillation – Characterization of the Arrhythmogenic Electrogram Patterns During Atrial Fibrillation and Sinus Rhythm
Source: Sci Rep. 2020 Jun 4;10:9147. doi: 10.1038/s41598-020-65564-2 (PMC7272441; doi:10.1038/s41598-020-65564-2)
Supplement: Supplementary file 4 — Supporting information. [file 41598_2020_65564_MOESM4_ESM.docx]

SUPPORTING INFORMATION

**Specific Electrogram Characteristics Identify the Extra-Pulmonary Vein Arrhythmogenic Sources Maintaining Persistent Atrial Fibrillation – Characterization of the Arrhythmogenic Electrogram Patterns During Atrial Fibrillation and Sinus Rhythm**

**Short Title: Electrogram Characteristics at Arrhythmogenic Sources in Atrial Fibrillation**

^1,^*Amir Jadidi, MD, ^2,^*Mark Nothstein, MSc, ^1^Juan Chen, BSc, ^1^Heiko Lehrmann, MD, ^2^Olaf Dössel, PhD, ^1^Jürgen Allgeier, MD, ^3^Dietmar Trenk, MD, ^4^Franz-Josef Neumann, MD, ^2^Axel Loewe, PhD, ^1,^*Björn Müller-Edenborn, MD, ^1,^*Thomas Arentz, MD

*These authors contributed equally to this manuscript

^1^Department of Electrophysiology, University-Heart-Center Freiburg-Bad Krozingen, Bad Krozingen Campus, Germany

^2^Institute of Biomedical Engineering, Karlsruhe Institute of Technology, Karlsruhe, Germany

^3^Department of Clinical Pharmacology, University-Heart-Center Freiburg-Bad Krozingen, Bad Krozingen Campus, Germany

^4^ Department of Cardiology, University-Heart-Center Freiburg-Bad Krozingen, Bad Krozingen Campus, Germany

**Corresponding author:**

Amir Jadidi, MD

University-Heart-Center Freiburg-Bad Krozingen

Südring 15

79189 Bad Krozingen

Germany

Phone: +49 7633 402 4334

Email: amir.jadidi@universitaets-herzzentrum.de


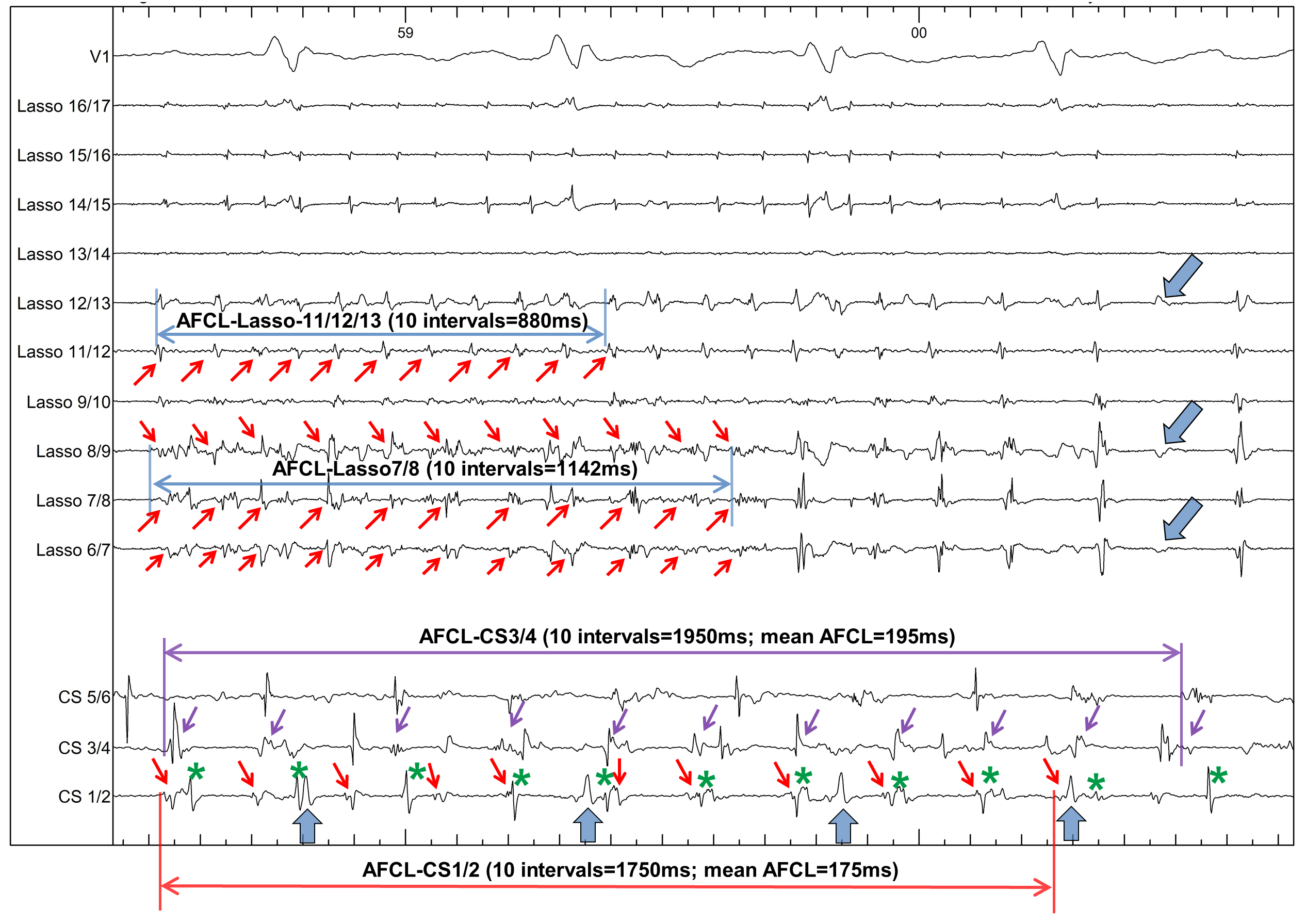


SUPPLEMENTAL FIGURE 1


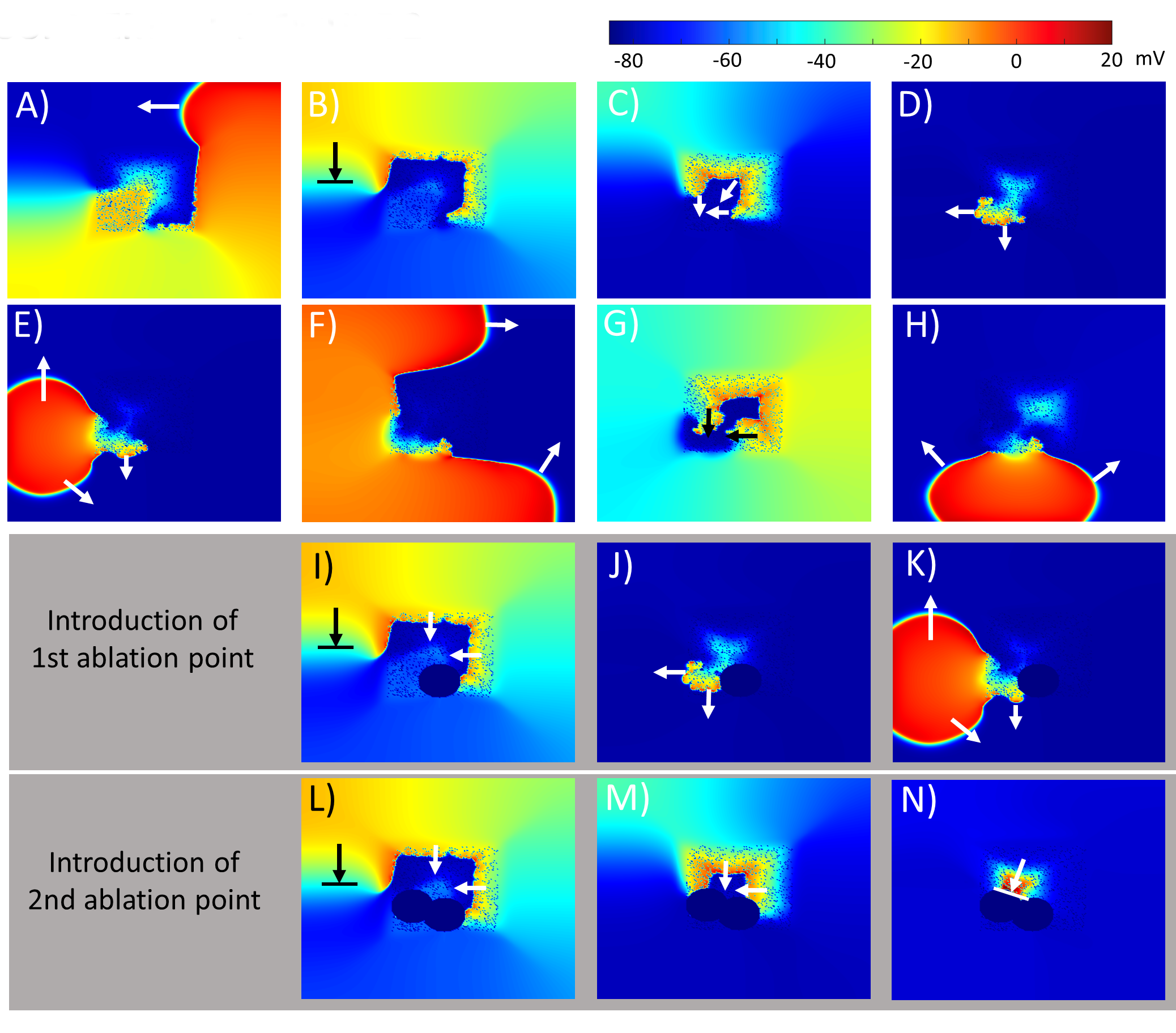


SUPPLEMENTAL FIGURE 2
